# Supplementary material for: The Amino Acid-mTORC1 Pathway Mediates APEC TW-XM-Induced Inflammation in bEnd.3 Cells
Source: Int J Mol Sci. 2021 Aug 26;22(17):9245. doi: 10.3390/ijms22179245 (PMC8431488; doi:10.3390/ijms22179245)
Supplement: Supplementary file 1 [file ijms-22-09245-s001.zip › ijms-1318476 supplementary.pdf]

Supplementary Material

Supplementary Table S1

List of oligo sequences for qPCR used in the study

Primers	Sequence (5'-3')	References
IL-6-F	TGCAAGAGACTTCCATCCAGT	Newly generated sequence
IL-6-R	GTGAAGTAGGGAAGGCCG	Newly generated sequence
IL-1β-F	ATGAAAGACGGCACACCCAC	Newly generated sequence
IL-1β-R	GCTTGTGCTCTGCTTGTGAG	Newly generated sequence
TNF-α-F	AGGCACTCCCCCAAAAGAT	Newly generated sequence
TNF-α-R	TGAGGGTCTGGGCCATAGAA	Newly generated sequence
iNOS-F	CAGCTGGGCTGTACAAACCTT	Newly generated sequence
iNOS-R	CATTGGAAGTGAAGCGTTTCG	Newly generated sequence
SLC1A1-F	GCCAGTTACATTCCGCTGTGCG	[57]
SLC1A1-R	TGCAGACCATGACCCGAGAGCA	[57]
SLC1A2-F	CAAGTCTGAGCTGGACACCA	[57]
SLC1A2-R	GTGTGCGGCATAGACACACT	[57]
SLC1A3-F	TTGCAGCAAGGGGTCCGCAA	[57]
SLC1A3-R	GCAGTGACCGTGAGCAGAACGA	[57]
SLC1A4-F	TGCCGCTGGGTTAAAGGATT	Newly generated sequence
SLC1A4-R	CAGCGCGTGAGTTTATTGGG	Newly generated sequence
SLC1A6-F	AGAAAAGGGGGCATCAAGGG	Newly generated sequence
SLC1A6-R	CGAGCACACAAATGCAACAGT	Newly generated sequence
SLC1A7-F	GTGGCCTGCTCTAATTTCCCA	Newly generated sequence
SLC1A7-R	CTGTTGGCACCATTCCATGC	Newly generated sequence
SLC6A14-F	TGGCTATGCAGTGGGATTGG	Newly generated sequence
SLC6A14-R	GCCCACGGCAATACACTTTG	Newly generated sequence

SLC7A9-F	TGGTGGTCAGCTGAGAGAGT	Newly generated sequence
SLC7A9-R	TATGATGAGACAGGGCCCCA	Newly generated sequence
SLC7A10-F	GCTCGTGGGTGACACATACA	Newly generated sequence
SLC7A10-R	CATGGGGCCATTTTCCTCCT	Newly generated sequence
SLC7A11-F	AATACGGAGCCTTCCACGAG	Newly generated sequence
SLC7A11-R	ACTGTTCGGTCGTGACTTCC	Newly generated sequence
SLC38A1-F	AGCACAGGCGACATTCTCATC	[58]
SLC38A1-R	ACAGGTGGAACTTTGTCTTCTTG	[58]
SLC38A2-F	ACAAATGGGTTGTGGTATCTG	[58]
SLC38A2-R	CCTAGATTTCTCAGCAGTGACAATG	[58]
SLC38A4-F	GTTCAGGAAAGACGGCACAG	Newly generated sequence
SLC38A4-R	GGTGAACCGAGTAGAGCGAG	Newly generated sequence
GAPDH-F	ACAACTCACTCAAGATTGTCAGCA	[58]
GAPDH-R	ATGGCATGGACTGTGGTCAT	[58]

Supplementary Table S2

List of oligo sequences for siRNA and overexpression DNA used in the study

Primers	Sequence (5'-3')	References
Slc38a2-mus1	CUGCCAUGCUGAUCUUUAUTT<br>AUAAAGAUCAGCAUGGCAGTT	Newly generated sequence
Slc38a2-mus2	CACGCUAUUUCAUCUUCAATT<br>UUGAAGAUGAAAUAGCGUGTT	Newly generated sequence
Slc38a2-mus3	GGCUAAUACUGGAAUUGCUTT<br>AGCAAUUCCAGUAUUAGCCTT	Newly generated sequence
Slc1a6-mus1	GCAUCCUAAUGGUUACCAUTT<br>AUGGUAACCAUUAGGAUGCTT	Newly generated sequence
Slc1a6-mus2	GCAGGAGGUGAUAAGCUUUTT<br>AAAGCUUAUCACCUCCUGCTT	Newly generated sequence
Slc1a6-mus3	GCUGGAAAGAUUCUGGAAATT<br>UUUCCAGAAUCUUUCCAGCTT	Newly generated

		sequence
mTOR-mus1	CCACCAGAAUUGGCAGAUUTT<br>AAUCUGCCAAUUCUGGUGGTT	Newly generated sequence
mTOR-mus2	GCAAAGACCUCAUGGGCUUTT<br>AAGCCCAUGAGGUCUUUGCTT	Newly generated sequence
mTOR -mus3	GCUCACUGCUGUGCUCUAUTT<br>AUAGAGCACAGCAGUGAGCTT	Newly generated sequence
Control-mus	UUCUUCGAACGUGUCACGUTT<br>ACGUGACACGUUCGGAGAATT	Newly generated sequence
Slc38a2-CF	CTCGAGATGAAGAAGACCGAAATGGGAAG	Newly generated sequence
Slc38a2-CR	GTCGACTTAGTGCCCACCTGCAG	Newly generated sequence
Slc1a6-CF	CTCGAGATGAGCAGCCACGGCAAT	Newly generated sequence
Slc1a6-CR	GTCGACTCACATGACGCTCTCATTACCTC	Newly generated sequence

Supplementary Table S3

List of oligo sequences for qPCR used in the study

Name	Source	Catalog number
aniti-SNAT2 (C-6)	Santa Cruz Biotechnology, Santa Cruz, CA, USA	sc-393528
anti-EAAT4	Abcam, Cambridge, UK	ab41650
anti-mTOR (7C10)	Cell Signaling Technology, Danvers, MA, USA	2983T
anti-phospho-mTOR (Ser2448)	Cell Signaling Technology, Danvers, MA, USA	2971S
anti-phospho-p70S6K (Thr421/Ser424)	Cell Signaling Technology, Danvers, MA, USA	9204S
anti-phospho-4E-BP (Thr37/46)	Cell Signaling Technology, Danvers, MA, USA	2855T
anti-GAPDH	Cell Signaling Technology, Danvers, MA, USA	2118S

Supplementary Figures

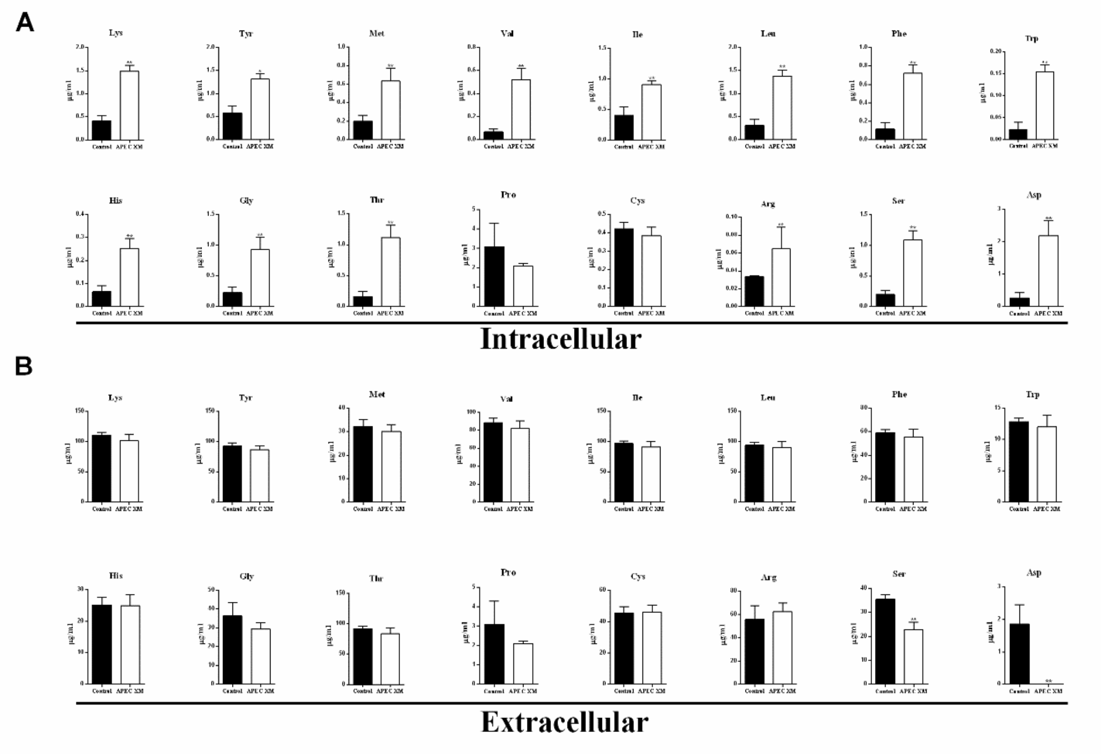

Supplementary Figure S1: APEC XM infection induced an increase in the intracellular content of other 14 amino acids but not affect the content of other 14 amino acids in the supernatant at 1 h postinfection. **(A)** The intracellular content of other 16 amino acids. **(B)** The extracellular content of other 16 amino acids. Data are from one experiment representative of three independent experiments, with six replicates per group. Data represent means ± SD. *$p < 0.05$, **$p < 0.01$.

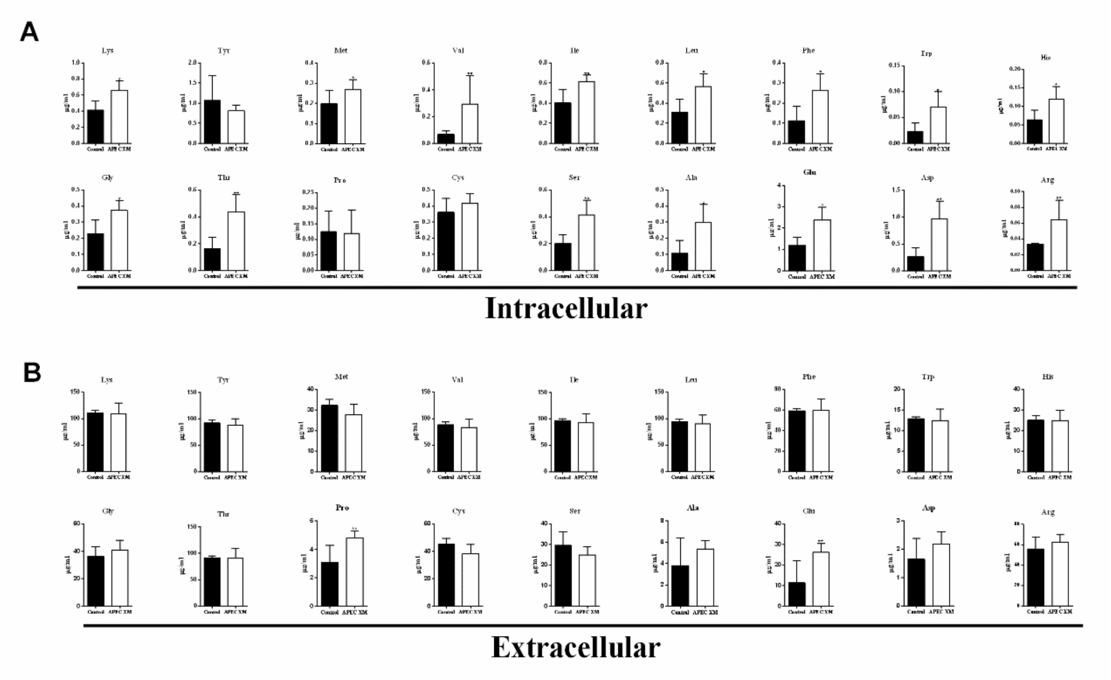

Supplementary Figure S2: APEC XM infection induces an increase in the intracellular content of 14 amino acids, but not affects the extracellular content of 18 amino acids at 12 h postinfection. **(A)** The intracellular content of 18 amino acids. **(B)** The extracellular content of 18 amino acids. Data are from one experiment representative of three independent experiments, with six replicates per group. Data represent means ± SD. **p* < 0.05, ***p* < 0.01.
